# Advancing PPG Signal Quality and Know-How Through Knowledge Translation—From Experts to Student and Researcher

**DOI:** 10.3389/fdgth.2020.619692

**Published:** 2020-12-21

**Authors:** Samuel Huthart, Mohamed Elgendi, Dingchang Zheng, Gerard Stansby, John Allen

**Affiliations:** ^1^Faculty of Medical Sciences, Newcastle University, Newcastle upon Tyne, United Kingdom; ^2^Department of Obstetrics and Gynaecology, University of British Columbia, Vancouver, BC, Canada; ^3^Research Centre for Intelligent Healthcare, Coventry University, Coventry, United Kingdom; ^4^Northern Vascular Centre, Freeman Hospital, Newcastle upon Tyne, Unite Kingdom; ^5^Northern Medical Physics and Clinical Engineering Department, Freeman Hospital, Newcastle upon Tyne, United Kingdom

**Keywords:** digital health, photoplethysmography, pulse, peripheral arterial disease, pulse wave analysis, signal quality, wearable sensor, artefact rejection

## Abstract

**Objective:** Despite the vast number of photoplethysmography (PPG) research publications and growing demands for such sensing in Digital and Wearable Health platforms, there appears little published on signal quality expectations for morphological pulse analysis. Aim: to determine a consensus regarding the minimum number of undistorted i.e., diagnostic quality pulses required, as well as a threshold proportion of noisy beats for recording rejection.

**Approach:** Questionnaire distributed to international fellow researchers in skin contact PPG measurements on signal quality expectations and associated factors concerning recording length, expected artifact-free pulses (“diagnostic quality”) in a trace, proportion of trace having artifact to justify excluding/repeating measurements, minimum beats required, and number of respiratory cycles.

**Main Results:** 18 (of 26) PPG researchers responded. Modal range estimates considered a 2-min recording time as target for morphological analysis. Respondents expected a recording to have 86–95% of diagnostic quality pulses, at least 11–20 sequential pulses of diagnostic quality and advocated a 26–50% noise threshold for recording rejection. There were broader responses found for the required number of undistorted beats (although a modal range of 51–60 beats for both finger and toe sites was indicated).

**Significance:** For morphological PPG pulse wave analysis recording acceptability was indicated if <50% of beats have artifact and preferably that a minimum of 50 non-distorted PPG pulses are present (with at least 11–20 sequential) to be of diagnostic quality. Estimates from this knowledge transfer exercise should help inform students and researchers as a guide in standards development for PPG study design.

## Introduction

Photoplethysmography (PPG) is a vascular optical measurement technique, used to detect blood volume changes in the microvascular bed of target tissue (1). Many studies have been conducted investigating various body sites as a single measurement (e.g., single PPG sensor located on a single body site) and multi-site measurements (multiple PPG sensors located across a range of body sites). The finger and toe pad sites are usually assessed. A range of features of the pulse wave have been studied, including pulse transit time, pulse interval, peak-to-peak interval, amplitude, pulse contour, as well as their natural variability (1). Subsequently, PPG has been utilized in an array of settings, from bedside physiological measurement e.g., heart rate, oxygen saturation, to hypertension assessment, and detailed peripheral vascular assessment (1–4). PPG has also become a key sensing technology in Digital and Wearable Health devices.

**Figure 1:**
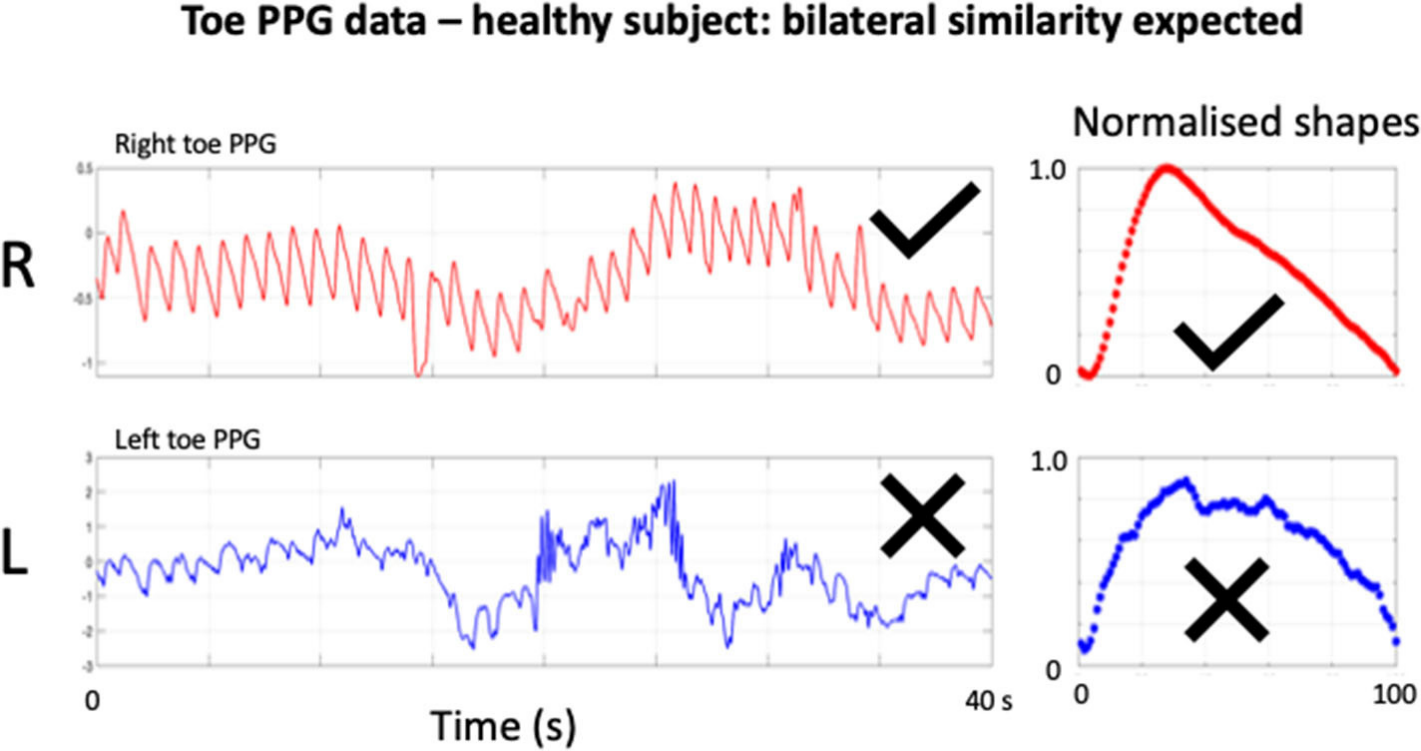
Example of bilateral toe PPG recording from a healthy subject. “AC” pulsatile components can be seen for the toe recording, each superimposed on a slowly varying “DC” baseline. Bilateral similarity would be expected for the pulse features at the toes, including for normalized shape averaged over all beats present. The contralateral right (R) toe trace is of reasonable quality and has a normal shape characteristic. However, there is unacceptable levels of artifact present in the left (L) toe trace and appears to result in an abnormal damped normalized pulse shape. Attempts at further signal filtering and/or cluster analysis would be unlikely to salvage the left side toe trace for classification purposes.

It is well-established that there is variability in the PPG waveform over time and that there can be differences in morphology and dynamics between different peripheral body sites (1), for example respiration as well as blood pressure changes can modulate PPG signals over periods of seconds/10's of seconds. Furthermore, artifact from sensor and limb movement and/or tremor can limit the reliable extraction of pulse features and so having a recording of sufficient length helps identify, and thus reject such episodes of noise (Figure 1) (5). These signal variability considerations form the rationale behind taking an average of multiple beats for representative morphological analysis of the PPG “AC” pulsatile component and the motivation for this study. However, despite the broad range of studies concerning applications of PPG especially in Digital Health (1, 3), as well as the significantly smaller number of investigations focussed specifically on quantifying signal quality (6–12), there appears little yet published on signal quality expectations, e.g., minimum length of recording or proportion of noisy beats needed to reject a recording, which consequently affects reproducibility and ultimate measurement value. We therefore carried out a consensus exercise by composing a questionnaire. We aimed to determine for morphological pulse analysis if there was agreement regarding minimum recording length, the minimum number of undistorted i.e., diagnostic quality pulses required, as well as the threshold proportion of noisy beats needed for the recording. Our wider goal is to transfer knowledge from experienced PPG workers to other (future) researchers and students internationally.

## Methods

We developed a set of questions based on a specific clinical PPG measurement scenario in our questionnaire: finger and toe pad PPG measurements carried out on a healthy adult subject, acclimatized for at least 10 min within a warm room. The individual would be relaxed, lying supine with their arms by their side, having been instructed to lie still and breathe gently throughout. We identified a group of selected fellow PPG workers established in the field and all of those contacted were known to have published/presented their research, encompassing various fields in the cardiovascular application of PPG. The questionnaire was distributed internationally to known fellow researchers working in skin contact PPG measurements.

The 6 questions on the questionnaire applicable to morphological analysis of the “AC” pulsatile component of PPG, i.e., pulse shape (13, 14), are summarized in the Appendix. Each question had specific numerical/range selections to choose from, there were no open responses to these questions. The questions related to the minimum length of recording time, the expected proportion of good quality beats per recording, the extent of noise needed to reject a recording, the minimum number of total and also sequential non-distorted i.e., diagnostic quality pulses required (criteria for an analysable beat outlined by Orphanidou (9): pulse requiring a clear peak, trough, anacrotic, and catacrotic phases), as well as the minimum number of respiratory cycles per recording needed, noting the low-frequency alterations in the PPG signal associated with respiration (15). The results from a subset of participants specializing in PPG and peripheral arterial occlusive disease (PAOD) diagnostics were also noted. Two authors independently checked the results from the survey.

## Results

In total, we approached 26 individuals *via* e-mail worldwide. Eighteen agreed to take part and give their views in the poll, coming from organizations in countries covering at least 3 continents (i.e., including the US, Europe and Asia). One questionnaire was only partially completed, and thus removed from the study—giving 17 responses in total to summarize.

With the exception of question 1, participants could select an answer relating to measurements at finger and toe sites independently. However, due to differences in experience, some opted to answer for only one measurement site. Hence, there was a discrepancy in the number of responses for the two body site locations (noting there were twelve full responses about the toe measurement site). In the few circumstances where participants had highlighted more than one answer per question for a single site, we decided to use their lowest answer, as this indicated the minimum threshold that they deemed acceptable.

**Figure 2:**
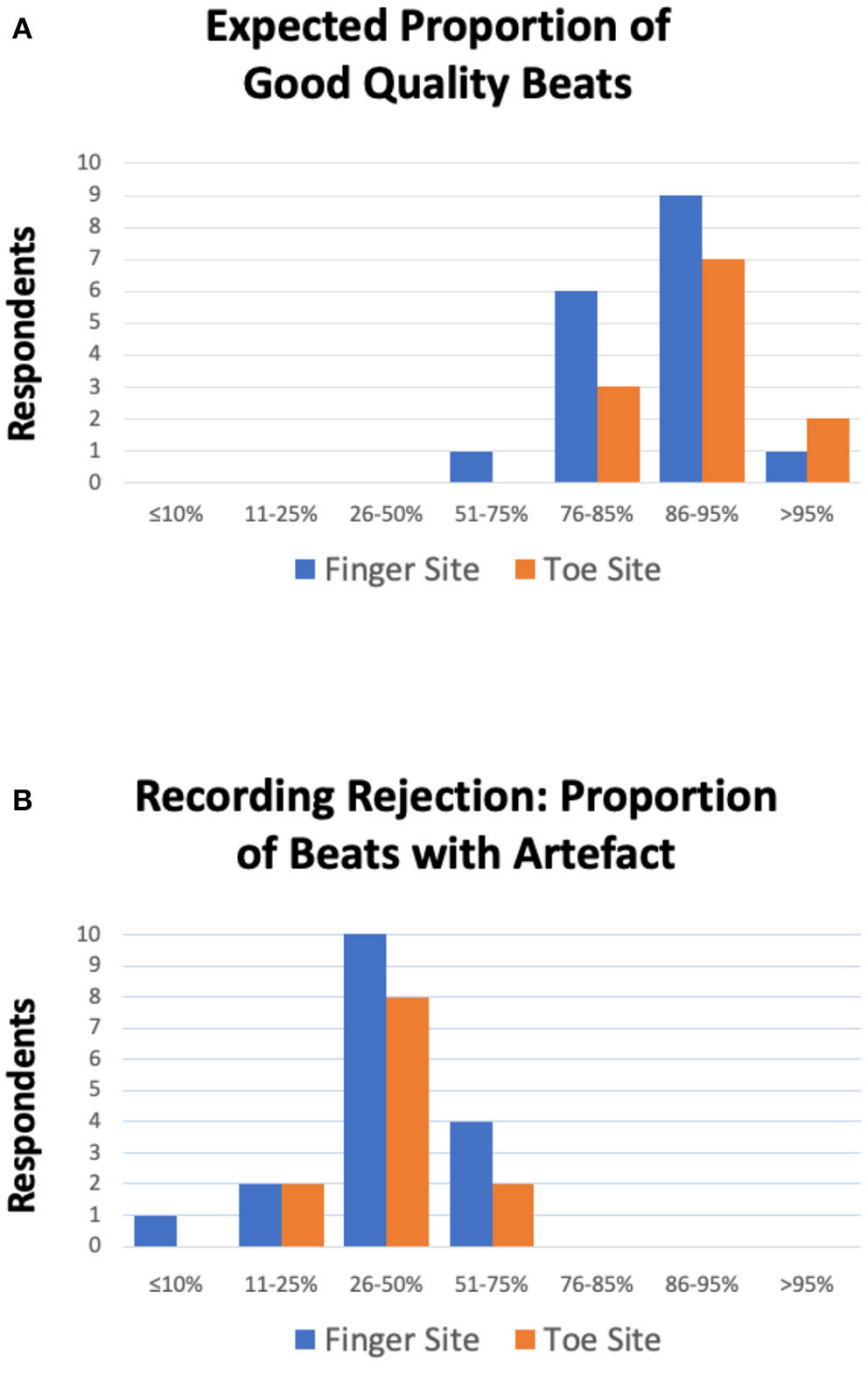
**(A)** Expected proportion of good quality beats i.e., artifact free beats per PPG recording. For both finger (blue) and toe (orange) measurement sites, all participants expected the majority of the recording to be undistorted i.e., of diagnostic quality (>51%). Furthermore, the majority of respondents expected ≥86–95% of beats to be of good quality (*n* = 9 and *n* = 7 respondents for the finger and toe sites, respectively). **(B)** Proportion of beats with artifact required to reject a PPG recording for the finger (blue) and toe (orange) sites. For both sites, the majority of respondents advocated that ≥26–50% of beats having noise was sufficient to reject a recording (*n* = 10 and *n* = 8 respondents for the finger and toe sites, respectively).

The responses did tend to vary across the survey, but we were able to obtain a modal range value for each of the related questions (Table 1). For morphological analysis, for both the finger and toe sites, a minimum recording time of 2 min was a recommended target and participants expected 86–95% of a PPG recording to be of diagnostic quality i.e., beats with no distortion. Participants advocated a threshold for the proportion of beats with artifact required to reject a recording in the range 26–50%, and a minimum number of sequential undistorted beats of 11–20. Less clear patterns from respondents, due to spread, were for the number of respiratory cycles (modal choice was marginally for >10 cycles but with responses similar for 2 and 5 cycles selections) and the minimum number of total undistorted beats (for both finger and toe sites the modal selection was marginally for 51–60 beats). Figure 2 shows key consensus results for the expected proportion of good quality pulses and for the proportion of beats with artifact to reject a recording. As well as such bar charts, a series of scatterplots were also produced in an attempt to identify relationships between answers to different questions, however no obvious correlations were observed and have therefore not been included.

**Table 1:** Summary of modal selections for morphological PPG pulse wave analysis.

	**Finger site**	**Toe site**
Modal selection for minimum PPG recording time (minutes)	2	2
Modal selection of range for expected proportion of undistorted i.e., diagnostic quality beats (%)	86–95	86–95
Modal selection of range for proportion of beats with artifact to reject a recording (%)	26–50	26–50
Modal selection of range for minimum number of undistorted i.e., diagnostic quality beats in the recording	51–60	51–60
Modal selection of range for minimum number of successive undistorted i.e., diagnostic quality beats in the recording	11-20	11-20
Modal selection for minimum number of respiratory cycles	>10	>10

*The modal selections on the questionnaire were similar overall for finger and toe measurement sites*.

The results from a subset of participants specializing in toe PPG and PAOD diagnostic assessments showed, however, only 4 of the 17 responding authors had published data for this application area. Nevertheless, when the answers pertaining to these specific parameters were compared to the rest of the respondents, there appeared general agreement.

## Discussion

Responses varied across the survey, although for most questions/sites a modal value could be ascertained. This work pointed us to a potential memorable 50/50 rule of thumb for guidelines in PPG i.e., finger and toe PPG pulse traces of a few minutes in length can be acceptable for morphological pulse wave analysis provided there was <50% of the beats with artifact and that a minimum of 50 undistorted i.e., diagnostic quality beats were present, and that there is potentially an additional need for at least 10–20 successive good quality beats in the recording. Given the vastness of PPG application, there is unfortunately no exact way to formulate a “one size fits all” standard but our results should have value in helping in knowledge transfer to guide researchers and medical device developers in PPG measurement and analysis.

The broadness in survey responses we believe was in part related to a lack of agreed guidelines in PPG measurement protocol and signal quality expectations. Coupled with this, in relation to wider measurement protocol considerations, there is an absence of standardized equipment (e.g., key probe/clip attachment designs) and measurement set-up, other than the general consensus that participants should be allowed a period of time to rest (but no standardized time known), and to acclimatize to the ambient measurement conditions (but no standardized temperature and/or humidity defined) (1). Future follow-up surveys should consider seeking expert consensus in each of these areas for the benefit of wider measurement communities. There is also a large number of clinical applications covered by those surveyed and future studies could certainly look at the applications aspect in more detail with specific analysis and visualization of results in mind. Additionally, another possible reason for the lack of a general recommendation, is a current focus on noise-reducing and waveform identifying algorithms (16), an important area of study especially concerning the commercial use of PPG in wearable sensor systems for modern Digital Healthcare. If the developed algorithms can robustly eliminate noise and extract analysable pulse waves unfailingly, one might argue there would be no need for a standard, as the remaining signal should be of good quality. However, developing noise-reducing algorithms in PPG has proved to be difficult, for example with clustering methods ineffective with PPG signals dominated by noise (17). Furthermore, excessive filtering often leads to distortion of the PPG pulse shape, masking useful physiological information, whilst too little filtering may allow the quasi-static “DC” component to dominate over the “AC” pulsatile component (1). In PPG this “DC” component can include the low / very low frequency changes of the signal and not just be a value at 0 Hz (1). The high pass filter being particularly important in PPG and needs careful selection for PPG shape assessment, with 0.15–0.2 Hz proposed by Allen and Murray (18) showing no clear shape distortion for multi-site PPG measurements at ear, finger and toe sites in a group of healthy subjects (18). This work from 2004 also showed that the ratios for PPG “DC” to “AC” components were similar at finger and toe sites for this range of high pass filter settings. This “DC”:“AC” measure of PPG variability also links to survey findings that responses were similar for finger and toe sites overall. To reiterate, the measurement methods and protocol used are very important and should always be clearly defined. For example, measurement device parameters, including the filtering, operating wavelength, and mode used, should be declared in research publications concerning contact PPG.

It is also important to comment on our bias assessment of the survey design and subsequent analysis. A survey may include a form of error such as sampling variability, interviewer effects, frame errors, response bias, and non-response bias (19–22). We designed the survey questions on PPG morphology and signal quality to contain only close-ended questions, which are answered by a simple selection from 7 (8 for Q1) choices. The main reason being to create data that are easily quantifiable, and straightforward to code. This also allowed us to categorize respondents into groups based on the options they have selected, thus increasing interpretability of the data. We also minimized all biases in our research with the following areas considered. Specification error—this did not occur as there was clear communication between the experts and data analysts. Frame error—this did not occur as our target population was those experienced in PPG research. Non-response error—in our survey all experts responded to questions regarding the finger site, however 12 (of 17) responded to the questions concerning the toe sites, potentially leading to a non-response error in this scenario. We suggest that it was the case that the 5 non-respondents, pertaining to toe measurements, focused their answers on their main measurement site of interest rather than guessing for a site they had little experience of. We also note that we did not add any value on behalf of any of the experts. Measurement error—our survey was carefully and manually carried out to reduce the risk of such an error. Finally, processing error—the lead (SH) and corresponding (JA) co-authors independently checked all results presented in this paper from the original set of questionnaire responses.

To our knowledge, this is the first report of its kind drawing on the knowledge and experience of published PPG workers. We obtained survey responses from 17 (12 of these responded on toes) well-respected experts in the field, noting that in Delphi exercises, a minimum of 12 respondents is generally considered to be sufficient to enable consensus to be achieved (23, 24). A possible limitation though may be the varying experience among the researchers contacted, albeit working predominantly in morphological analysis, but with lesser involvement at the toe site compared to the finger site. All of those contacted to take part were professionally known by the senior author (JA) and were known to have published/presented their research, encompassing various fields in the cardiovascular application of PPG across the globe. We accept that such PPG researcher choice would involve bias and is potentially a limitation of this study, and future wider surveys could be designed to assess differences in opinion between researchers from different geographical regions. However, this we believe is something for the future in a carefully designed survey setting out to investigate this specific aspect. Another limitation is the proposed ranges for some questions being wide, and thus being heavily open to interpretation, specifically the minimum length of time per recording.

Our results have shown value and indicated key recommendations for contact PPG recordings in relation to noise and signal quality expectations. It is also important to note signal quality and noise rejection are very important topics in clinical physiological measurement not just for PPG but for a range of signal types—this should be a very important topic for design of noise reduction algorithms in Digital Healthcare platforms including wearable sensing applications.

A potential future study would be to distribute another questionnaire investigating a selection of these suggested ranges further with narrower limits, and a more detailed description of the purpose of the measurement i.e., it may be that a large proportion of participants recommend that a recording warrants rejection if 41–50% noise is present, compared to 31–40%. Also, morphological analysis covers a wide range of sub-studies in PPG, and therefore many researchers would put forward answers to the above questions differing entirely, depending on the pulse feature in question. The specific application to Digital Health and Wearable sensing with PPG should be an added focus. Furthermore, even though individuals were given the opportunity to provide additional details at the conclusion of the survey, written justification for their decisions would have been insightful. In future follow-on surveys, a wider range of body sites should be considered (including the ear and forehead sites for example), as well user experiences and expectations with various forms of transmission and reflection mode sensor/tissue attachment technology as well as remote i.e., imaging PPG. Lastly, we believe it would next be helpful to issue the survey to an open biosensor-based research community, for future reference.

## Summary

In summary, our results can be used as a guide for future studies in PPG and especially morphological pulse wave analysis, specifically in determining wider views in justifying which signals to utilize, discard and repeat. This area is very important in Digital Healthcare systems with wearable sensing and the need to gather repeatable and meaningful PPG data. Our study also provides initial recommendations available for other workers in the field of PPG—facilitating knowledge transfer to students and researchers to support the move toward improved standardization in measurement protocol, morphological pulse wave analysis, as well as address the real-world problem of artifact reduction in PPG.

## Data Availability Statement

The raw data supporting the conclusions of this article will be made available by the authors, without undue reservation.

## Ethics Statement

The studies involving human participants were reviewed and approved by Newcastle University Ethics Committee (Online Application: Ref 3549/2020). The patients/participants provided their written informed consent to participate in this study.

## Author Contributions

SH, GS, and JA contributed to conception and design of the study. SH performed the statistical analysis and wrote the first draft of the manuscript. JA wrote sections of the manuscript and confirmed the reported analysis results. ME shaped the article's focus with formulating the knowledge translation aspect as well as supporting the bias assessments. All authors contributed to manuscript revision, read, and approved the submitted version.

## Conflict of Interest

Between 2014 and 2018 JA was the Chief Investigator on an NIHR i4i funded grant (II-C1-0412-20003) to develop a miniaturized version of multi-site PPG pulse vascular measurement technology—specifically for peripheral arterial disease detection in a primary care setting. The remaining authors declare that the research was conducted in the absence of any commercial or financial relationships that could be construed as a potential conflict of interest.

## Appendix

**Questionnaire Summary for PPG Morphological Analysis**

1. When obtaining a PPG trace, what would you recommend as a minimum duration of recording?
2. Given the specified measurement setting over a 2-min period, what percentage of beats would you expect to be of good quality?
3. At what percentage of noise, relative to the number of pulse waves acquired, should we discard a recording and/or repeat it?
4. In the exploration of the photoplethysmographic pulse shape, what would you recommend as a minimum number of diagnostic quality pulse waves, in order that the average obtained is a true representation of the individuals PPG pulse shape?
5. In the exploration of the photoplethysmographic pulse shape, what would you recommend as a minimum number of successive diagnostic quality pulse waves, in order that the average obtained is a true representation of the individuals PPG pulse shape?
6. What minimum number of respiratory cycles would you recommend recording a PPG trace for?
